# Return to Spontaneous Circulation in a Patient With Cholinergic Syndrome at the Emergency Department of Buea Regional Hospital, Cameroon

**DOI:** 10.1002/ccr3.71086

**Published:** 2025-10-01

**Authors:** Bertolt Brecht Kouam Nteungue, Berinyuy Nyuydzefon, Boris Arnaud Nteungue Kouomogne, Verla Vincent Siysi

**Affiliations:** ^1^ Anaesthesia and Intensive Care Unit Regional Hospital of Buea Buea Cameroon; ^2^ Department of Emergency Medicine, Anesthesia and Intensive Care, Faculty of Health Sciences University of Buea Buea Cameroon; ^3^ Department of Public Health Central University of Nicaragua Managua Republic of Nicaragua

**Keywords:** atropine, case report, cholinergic syndrome, emergency, herbal induce cholinergic toxicity, intensive care, organophosphate poisoning

## Abstract

With the increasing misuse of herbal treatment and pesticides, cases of cholinergic syndrome following herbal treatment or organophosphate poisoning have become increasingly common in our milieu. However, when advanced, mortality is high. We report the successful management of a case involving a 54‐year‐old African female with a known history of hypertension and people living with HIV/AIDS, who consumed a powdered concoction from a traditional herbalist with the aim of treating her hypertension. She was later found unconscious and brought to the Emergency Department. On arrival, clinical evaluation revealed an unconscious patient, gasping, with bradypnea and hypersalivation. A working diagnosis of cholinergic syndrome was made. She was intubated and treated with atropine and recovered one hour later, after which she was extubated and admitted to the intensive care unit (ICU) for supportive intensive care treatment. She was discharged 2 days after her admission to the ICU with counseling to take only medications prescribed by a medical doctor to avoid this situation in the future. Our case report shows that prompt and accurate therapy in the face of advanced organophosphate poisoning can lead to the rescue of life.

**Abbreviations**
AChE: acetylcholinesterase
HIV: human immunodeficiency virus
ICU: intensive care unit
IV: intravenous
OP: organophosphates
PAM: pralidoxime
PLHA: people living with HIV/AIDS
SpO2: peripheral oxygen saturation

**Summary**

- Cholinergic syndrome can rapidly lead to death if not promptly and adequately treated.
- In low‐income countries, it is often underdiagnosed, and there is insufficient data available on the condition.
- Our case report highlights the importance of early identification based on clinical evaluation and timely management.
- The use of airway protection and atropine, even in the absence of advanced resources, can be life‐saving, as demonstrated in our case.

## 1 Introduction

Cholinergic syndrome is a clinical toxidrome caused by overstimulation of acetylcholine (ACh) receptors, leading to a constellation of muscarinic, nicotinic, and central nervous system symptoms. Although organophosphates and carbamates are the most common causes, various herbs with cholinergic properties (acting as acetylcholine mimics or acetylcholinesterase inhibitors) can also induce this syndrome [1, 2].

Although developed countries have seen a decline in organophosphate poisoning cases due to stricter regulations on the use of these chemicals, developing countries continue to face clinical problems related to this issue in recent years. Pesticides are frequently used as a means of self‐harm due to their lethality and widespread availability in the developing world. Therefore, developing countries that rely heavily on agriculture and often have less stringent pesticide regulations account for the majority of organophosphate poisoning cases. Research indicates that deliberate poisoning results in a higher mortality rate than accidental exposure to these compounds [3].

Cholinergic toxicity is caused by substances that stimulate, enhance, or mimic the neurotransmitter acetylcholine, the primary neurotransmitter of the parasympathetic nervous system. Acetylcholine stimulates muscarinic and nicotinic receptors to cause muscle contraction and glandular secretions. Cholinergic toxicity occurs when too much acetylcholine is present at the receptor synapse, leading to excessive parasympathetic effects [4].

The mechanism involves excessive cholinergic receptor stimulation, which can be caused by substances that mimic, stimulate, or enhance acetylcholine. The symptom complex produced by the agent depends on what type of receptor or combination of receptors is activated. There are three types of cholinergic receptors: central, muscarinic, and nicotinic. Excess acetylcholine at muscarinic receptors will result in symptoms of increased secretions, bronchoconstriction, bradycardia, vomiting, and abdominal cramping. Excess acetylcholine at nicotinic receptors causes muscle fasciculations or paralysis due to activation of the neuromuscular junction. Acetylcholine excess in the central nervous system can cause confusion, headache, or drowsiness [5].

We report the case of successful management of cholinergic syndrome following the ingestion of a powdered concoction from a traditional herbalist.

## 2 Case History/Examination

A 54‐year‐old African female, known to be hypertensive and PLHA for over 17 years with compliance to her treatment, was brought by her children to the emergency unit for unresponsiveness, which began 15 min prior to arrival. Upon arrival, she was gasping with a respiratory rate of 3 breaths per minute, and oxygen saturation was undetectable with a pulse oximeter. The respiratory system showed normal vesicular breath sounds, equal air entry with no added sounds. There was no pulse, and she was hypersalivating. She was unconscious, with a Glasgow Coma Score of 3/15 (E1V1M1) and bilateral miosis unreactive to light.

### 2.1 The Diagnosis of Cholinergic Syndrom Was Made

Based on salivation, urination, bilateral miosis unreactive to light, and coma.

The predominant compound was muscarinic effects.

### 2.2 Differential Diagnosis

Herbals induce cholinergic toxicity.

Organophosphate poisoning.

Carbamate poisoning.

### 2.3 Investigations and Treatment

Emergency management constituted awake intubation with mechanical ventilation. Atropine 2 mg intravenous (IV) every 5 min for a total dose of 10 mg was administered. A bolus of 2 L of normal saline was given to treat hypotension. One hour later, there was a favorable response. The Glasgow Coma Score improved to 15/15 with persistent bilateral miosis, blood pressure of 151/86 mmHg, pulse rate of 112 bpm, and SpO2 of 98% with 5 L of oxygen through the endotracheal tube. Upon lung auscultation, she had bilateral rhonchi. She was extubated and admitted to the Intensive Care Unit for further care.

### 2.4 Initial Laboratory Tests Showed Results in Table 1

**TABLE 1:** Initial laboratory test.

Test	Results	Range	Remarks
Calcium	11.1	8.6–10.3 mg/dL	High
Chloride	112	98–108 mmol/L	High
Magnesium	2.8	1.9–2.5 mg/dL	High
Potassium	4.1	3.5–5.5 mmol/L	Normal
Sodium	146	135–145 mmol/L	High
C‐reactive protein	6	< 6 mg/L	High
Creatinine	1.1	0.7–1.4 mg/dL	Normal
Urea	23	12.8–42.8 mg/dL	Normal
White blood cells	13.10	3.5–9.5 10^9^/L	High
Lyphocytes	1.6	1.1–3.2 10^9^/L	Normal
Granulocytes	11.4	1.8–6.3 10^9^/L	High
Hemoglobin	15	11.5–15 g/dL	Normal
Platelets	550	125–350 10^9^/L	High
Fasting blood sugar	470	70–110 mg/dL	High

The patient was treated with ceftriaxone empirically for possible aspiration pneumonia, along with analgesics, proton pump inhibitors, and IV fluids.

When interviewed on day 2 of her hospitalization in the ICU, she reported that she had consumed a powdered treatment from a traditional healer for her hypertension for the first time the day before. She still exhibited tight bilateral myosis. Atropinization with atropine 1 mg every 3 min until her pupils returned to normal (for a total of 10 mg cumulatively) was administered. A chest X‐ray requested was normal.

She was discharged 2 days after her admission to the ICU and was advised to take only medications prescribed by a medical physician to avoid such a situation in the future.

## 3 Conclusion

Cholinergic syndrome is a life‐threatening condition, and early recognition is crucial for timely management and improved outcomes. While paraclinical assessments such as the dosage of toxic substances in urine, analysis of gastric lavage secretions, and cholinesterase levels are valuable for identifying the cause, they should not delay prompt therapy based on clinical judgment. In a resource‐limited setting, we successfully managed a case of advanced cholinergic syndrome due to swift advanced airway management and the availability of atropine.

## 4 Discussion

Our case represents a typical life‐saving intervention using purely clinical judgment in the absence of paraclinical workup. The powder consumed by the patient was not available for analysis. We did not test for the dose of toxic substances in urine or cholinesterase concentration due to unavailability in our context.

Herbal substances can induce cholinergic syndrome through several mechanisms:

- herbal substances can induce cholinergic syndrome e.g., pilocarpine, arecoline,
- pilocabition of acetylcholinesterase, resulting in increased acetylcholine levels (e.g., physostigmine, huperzine A, galantamine),
- physostigmine, huperzine A, galantamine sulting in increased acetylcholine levels (araccentral cholinergic effects).

Cholinesterase activity was not tested due to unavailability, the powder treatment took from the traditional healer was not present to analyze and identify the component responsible of cholinergic syndrome [2, 6].

Typically, the diagnosis of organophosphate poisoning is made based on the history of poisoning, the smell of pesticides, the characteristic clinical signs, and reduced cholinesterase activity. Measurement of plasma cholinesterase is useful for diagnosis, although it may not directly correlate with the severity of poisoning. Atropine remains the mainstay of treatment for organophosphate poisoning, with clear evidence of benefit if administered effectively. Atropine therapy should be monitored to maintain systolic blood pressure > 80 mmHg, pulse > 80 beats/min, and clear lung sounds on auscultation [7, 8, 9, 10]. Our patient exhibited signs of muscarinic syndrome, prompting us to initiate atropine treatment despite the absence of advanced diagnostic facilities.

The use of AChE reactivators is crucial in organophosphate poisoning, as atropine is not effective at the neuromuscular synapse. It is recommended that Obidoxime be started as a 250 mg IV bolus over 20–30 min, followed by a continuous infusion of 30 mg/h for 12–24 h until atropine is no longer needed and the patient has been extubated [7, 8]. However, caution should be exercised when using oximes in carbamate poisoning, as they can aggravate AChE inhibition [11]. An AChE reactivator was not used due to unavailability in our context.

Oxidative stress has recently been implicated as a factor in the mortality and morbidity induced by organophosphate (OP) compound poisoning. An overwhelming number of research papers are based on studies at the cellular and organ level [8]. Such studies have concluded that antioxidants can be used as adjunct compounds in the treatment of both chronic and acute OP poisoning. Still, the role of antioxidants in reducing the mortality and morbidity induced by OP compounds has scarcely been verified, as well as their role as adjunct treatment compounds for both structurally and functionally different OP compounds. The use of antioxidants remains controversial; some studies on animals and humans report no benefits against organophosphate poisoning [12, 13, 14]. We did not use them.

In resource‐limited settings, it is very challenging to differentiate cholinergic syndrome due to organophosphate poisoning from cholinergic syndrome due to herbal treatment. However, a management conducted only with atropine and supportive care with recovery of the patient may suggest another cause of cholinergic syndrome than OP poisoning where PAM is necessary to reverse AChE inhibition.

The availability of specific exams like Acetylcholinesterase activity, analysis of suspected agents inducing cholinergic syndrome, will avoid unnecessary drug administration like the use of pralidoxime for cholinergic syndrome where atropine can be enough like in our case.

## Author Contributions

**Bertolt Brecht Kouam Nteungue:** conceptualization, data curation, formal analysis, funding acquisition, investigation, methodology, project administration, resources, software, validation, visualization, writing – original draft, writing – review and editing. **Berinyuy Nyuydzefon:** supervision, writing – review and editing. **Boris Arnaud Nteungue Kouomogne:** writing – review and editing. **Verla Vincent Siysi:** methodology, project administration, supervision, validation, writing – review and editing.

## Ethics Statement

The authors have nothing to report.

## Consent

Written informed consent was obtained from the patient to publish this report in accordance with the journal's patient consent policy.

## Conflicts of Interest

The authors declare no conflicts of interest.

## Data Availability

The data that support the findings of this study are available from the corresponding author upon reasonable request.

## References

[1] D. P. Waller and B. F. Sampson , “Natural Products as Cholinergic Agents,” Journal of Pharmacy and Pharmacology 52, no. 7 (2000): 809–818.

[2] R. Wang , H. Yan , and X. C. Tang , “Progress in Studies of Huperzine A, a Natural Cholinesterase Inhibitor From Chinese Herbal Medicine,” Acta Pharmacologica Sinica 27, no. 1 (2006): 1–26. 10.1111/j.1745-7254.2006.00255.x 16364207

[3] E. L. Lott and E. B. Jones , “Cholinergic Toxicity,” in StatPearls [Internet] (StatPearls Publishing, 2024).

[4] E. L. Robb , A. C. Regina , and M. B. Baker , “Organophosphate Toxicity,” in StatPearls [Internet] (StatPearls Publishing, 2023). 29261901

[5] A. Vale , “Organophosphorus Insecticide Poisoning,” eJIFCC 11, no. 2 (1999): 30–35. 30720257 PMC6357250

[6] M. Heinrich and H. L. Teoh , “Galanthamine From Snowdrop—The Development of a Modern Drug Against Alzheimer's Disease From Local Caucasian Knowledge,” Journal of Ethnopharmacology 92, no. 2–3 (2004): 147–162. 15137996 10.1016/j.jep.2004.02.012

[7] M. Eddleston , N. A. Buckley , P. Eyer , and A. H. Dawson , “Management of Acute Organophosphorus Pesticide Poisoning,” Lancet 371 (2008): 597–607, 10.1016/S0140-6736(07)61202-1. 17706760 PMC2493390

[8] H. Thiermann , K. Kehe , D. Steinritz , et al., “Red Blood Cell Acetylcholinesterase and Plasma Butyrylcholinesterase Status: Important Indicators for the Treatment of Patients Poisoned by Organophosphorus Compounds,” Arhiv za Higijenu Rada i Toksikologiju 58 (2007): 359–366, 10.2478/v10004-007-0030-6. 17913691

[9] S. R. Bajracharya , P. N. Prasad , and R. Ghimire , “Management of Organophosphorus Poisoning,” Journal of the Nepal Health Research Council 14 (2016): 131–138, 10.33314/jnhrc.v14i3.868. 28327676

[10] A. M. King and C. K. Aaron , “Organophosphate and Carbamate Poisoning,” Emergency Medicine Clinics of North America 33 (2015): 133–151, 10.1016/j.emc.2014.09.010. 25455666

[11] T. Wille , L. Kaltenbach , H. Thiermann , and F. Worek , “Investigation of Kinetic Interactions Between Approved Oximes and Human Acetylcholinesterase Inhibited by Pesticide Carbamates,” Chemico‐Biological Interactions 206 (2013): 569–572, 10.1016/j.cbi.2013.08.004. 23962483

[12] S. X. Naughton and A. V. Terry, Jr. , “Neurotoxicity in Acute and Repeated Organophosphate Exposure,” Toxicology 408 (2018): 101–112, 10.1016/j.tox.2018.08.011. 30144465 PMC6839762

[13] N. Vanova , J. Pejchal , D. Herman , A. Dlabkova , and D. Jun , “Oxidative Stress in Organophosphate Poisoning: Role of Standard Antidotal Therapy,” Journal of Applied Toxicology 38 (2018): 1058–1070, 10.1002/jat.3605. 29516527

[14] S. M. Nurulain , P. Szegi , K. Tekes , and S. N. H. Naqvi , “Antioxidants in Organophosphorus Compounds Poisoning,” Arhiv a Higijenu Rada i Toksikologiju 64 (2013): 169–177, 10.2478/10004-1254-64-2013-2294. 23740557

